# Supplementary material for: Racah materials: role of atomic multiplets in intermediate valence systems
Source: Sci Rep. 2015 Oct 22;5:15429. doi: 10.1038/srep15429 (PMC4614842; doi:10.1038/srep15429)
Supplement: Supplementary Information [file srep15429-s1.pdf]

Supplementary Information for

Racah materials: role of atomic multiplets in intermediate valence systems

A. B. Shick,[1] L. Havela,[2] A. I. Lichtenstein,[3,4] and M. I. Katsnelson[5,4]

[1] *Institute of Physics, ASCR, Na Slovance 2, CZ-18221 Prague, Czech Republic*
[2] *Department of Condensed Matter Physics, Charles University, Ke Karlovu 5, CZ-12116, Prague, Czech Republic*
[3] *University of Hamburg, Jungiusstrasse 9, 20355 Hamburg, Germany*
[4] *Theoretical Physics and Applied Mathematics Department, Ural Federal University, Mira Str.19, 620002, Ekaterinburg, Russia*
[5] *Radboud University Nijmegen, Heyendaalseweg 135, 6525 AJ Nijmegen, The Netherlands*

PACS numbers: 71.28.+d, 71.20.-b

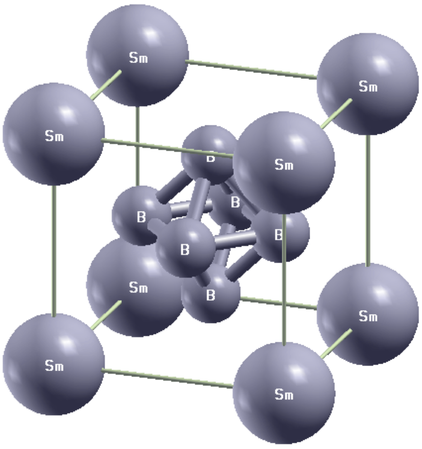

FIG. S1: The crystal structure of SmB_6.

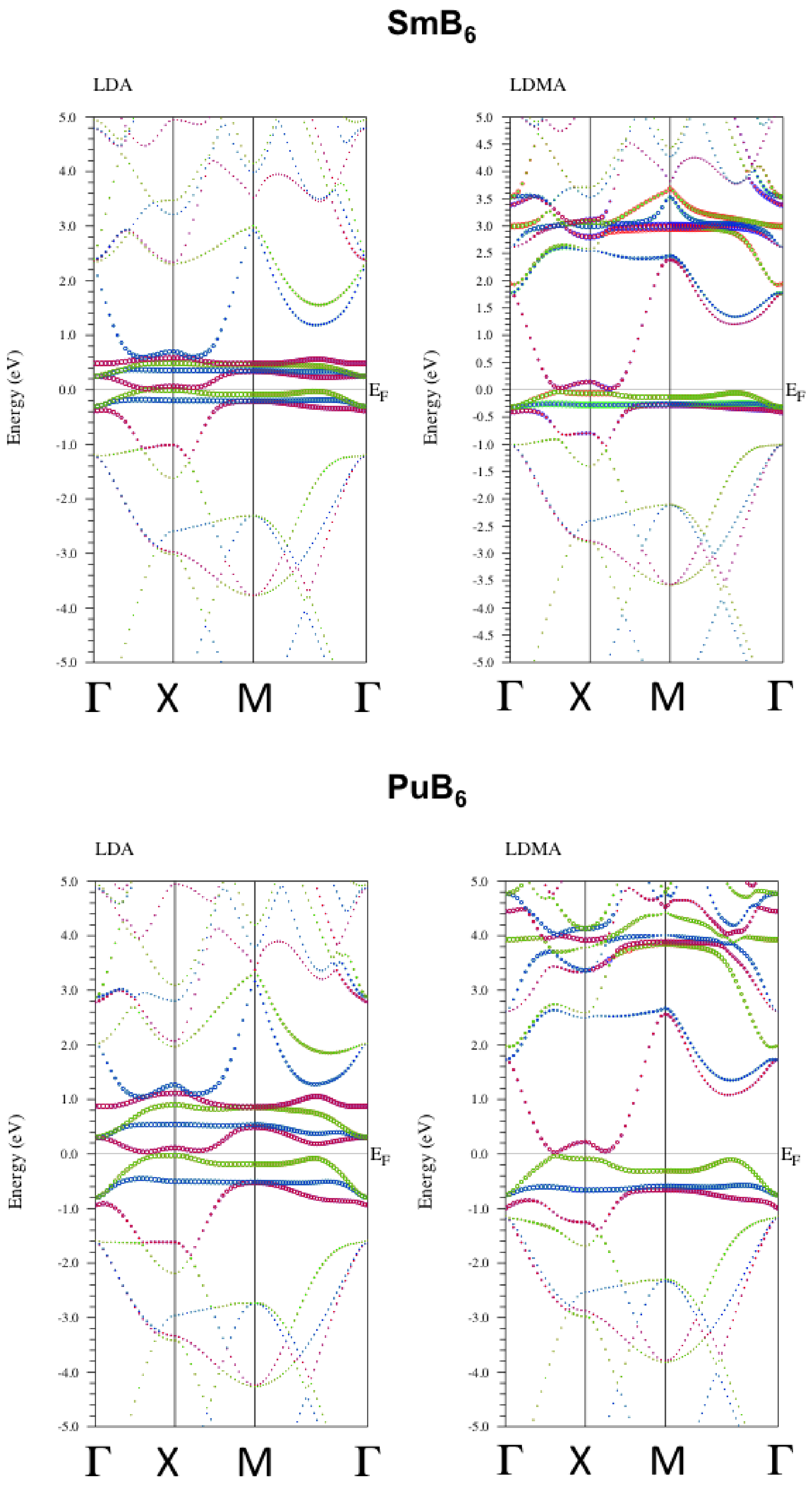

FIG. S2: SmB_6 (top) and PuB_6 (bottom) LDA and LDMA band structure.

In order to incorporate the dynamical self-energy effects into the LDMA band structure shown in Fig. 3 and Fig. S2, we make use of the first-order perturbation theory for the Green's function of the Eq. (3) with respect to $[\Sigma(z) - V_U]$, and write the $\mathbf{k}$-resolved spectral density $A(\mathbf{k}, z)$ as,

$$A(\mathbf{k}, z) = -\frac{Im}{\pi} \sum_n \Big[\frac{1}{z + \mu - \epsilon^n_{\mathbf{k}}} + \frac{\langle \Phi^n_{\mathbf{k}} | \, \Sigma(z) - V_U \, | \Phi^n_{\mathbf{k}} \rangle}{(z + \mu - \epsilon^n_{\mathbf{k}})^2} \Big]. \quad (1)$$

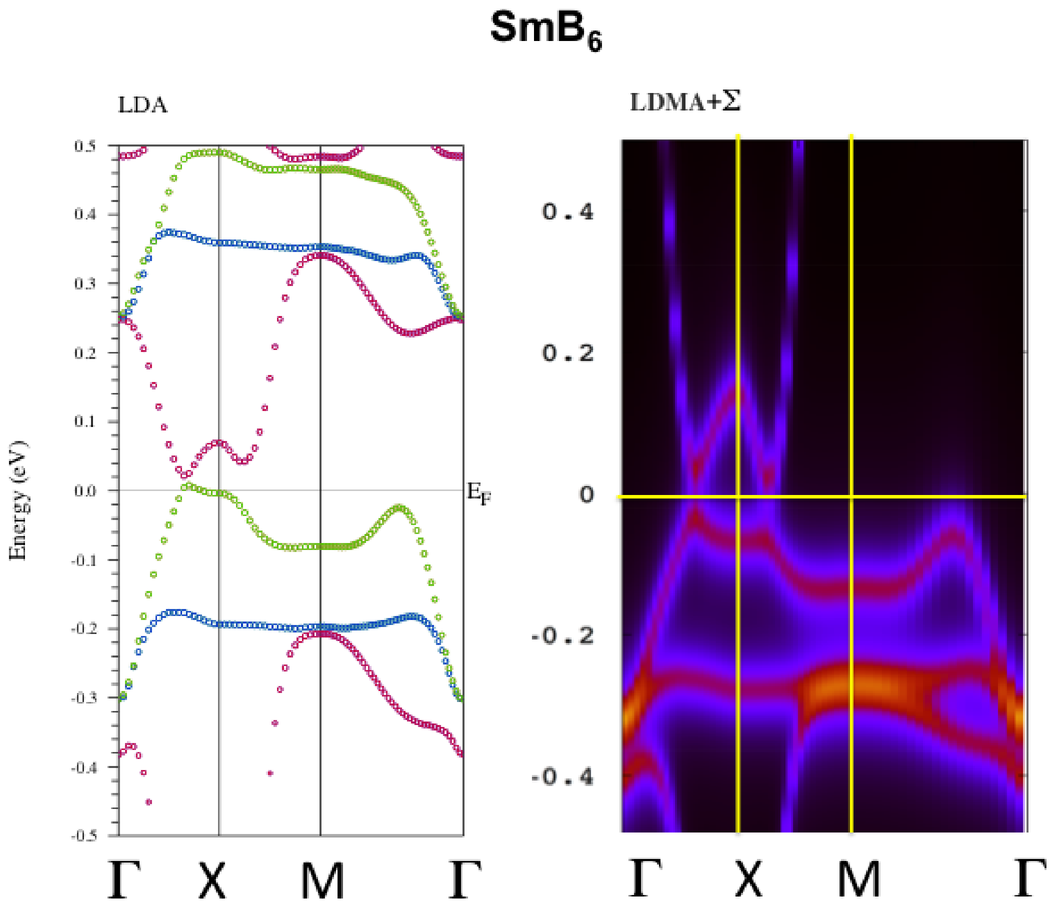

FIG. S3: SmB_6 LDA and LDMA + Σ band structure.

We plot in in Fig. (S3) together with the LDA band structure for SmB_6 . We notice that the effect of $\Sigma(z)$ on the LDMA band structure shown in Fig. 3(top) is rather small. The indirect band gap is somewhat reduced to 30 meV becoming closer to the experimental value of 20 meV. Also, it resembles some features of the DMFT calculated spectral DOS [1] as well as the experimental angular resolved photoemission (ARPES) [2]. Since the experimental ARPES is known be very surface sensitive, careful comparison with the theoretical calculations including the surface calculations is needed. These studies are beyond the scope of the present work, and left for the future.

[1] Junwon Kim *et al.,* Phys. Rev. B**90**, 075131 (2014).
[2] J. D. Denlinger *et al.,*, JPS Conf. Proc. **3**, 017038 (2014).
